# Fabrication techniques involved in developing the composite scaffolds PCL/HA nanoparticles for bone tissue engineering applications

**DOI:** 10.1007/s10856-021-06564-0

**Published:** 2021-08-11

**Authors:** Sivasankar Murugan, Sreenivasa Rao Parcha

**Affiliations:** Stem Cell Research Laboratory, Department of Biotechnology, National Institute of Technology, Warangal, Telangana 506004 India

## Abstract

A fine-tuned combination of scaffolds, biomolecules, and mesenchymal stem cells (MSCs) is used in tissue engineering to restore the function of injured bone tissue and overcome the complications associated with its regeneration. For two decades, biomaterials have attracted much interest in mimicking the native extracellular matrix of bone tissue. To this aim, several approaches based on biomaterials combined with MSCs have been amply investigated. Recently, hydroxyapatite (HA) nanoparticles have been incorporated with polycaprolactone (PCL) matrix as a suitable substitute for bone tissue engineering applications. This review article aims at providing a brief overview on PCL/HA composite scaffold fabrication techniques such as sol–gel, rapid prototyping, electro-spinning, particulate leaching, thermally induced phase separation, and freeze-drying, as suitable approaches for tailoring morphological, mechanical, and biodegradability properties of the scaffolds for bone tissues. Among these methods, the 3D plotting method shows improvements in pore architecture (pore size of ≥600 µm and porosity of 92%), mechanical properties (higher than 18.38 MPa), biodegradability, and good bioactivity in bone tissue regeneration.

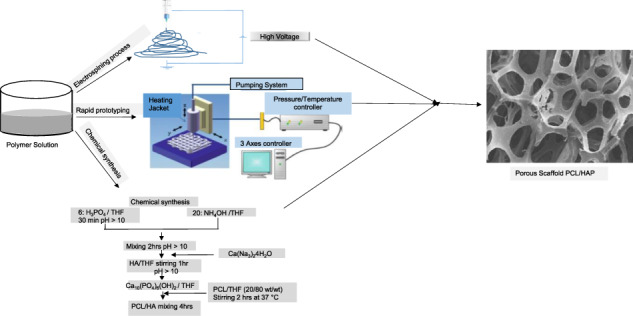

## Introduction

Bone is a rigid, complex, and hierarchical structure consisting of collagen and hydroxyapatite (HA), which provides hardness and toughness to the tissue. Bone tissue consists of two different structures: an outer cortical bone, with less than 10% porosity, and an inner cancellous bone, with a porosity of 50–90%. Both structures undergo dynamic remodeling, maturation, differentiation, and resorption that are controlled via interactions among osteocyte, osteoblast, and osteoclast cells [1]. In bone remodeling, osteoblasts are primarily responsible for a new bone formation, while osteoclasts are responsible for bone resorption which is a dynamic process for maintaining a healthy bone [2]. Bone-related defects can be caused by many conditions, including trauma, tumors, and bone diseases, which cannot heal by themselves. Tissue engineering emerges as a potential approach to overcome the challenges of conventional in bone graft treatments [3]. Bone tissue engineering strategies involve a combination of scaffolds, growth factors, and stem cells to restore the function of injured tissue and overcome the complications associated with bone and other tissues repairing [4–8]. The scaffold should meet specific characteristics, such as physico-chemical and mechanical properties, to achieve cell attachment, proliferation, and maturation, thereby enabling bone tissue formation [9–12]. The interconnected porous structure and pore size distribution are important factors to be considered for 3D scaffold fabrication, which contributes to cell penetration into the scaffold, and allowing an adequate colonization of the scaffold. A pore size with the high surface to volume ratio, as well as porosities, support cell attachment, proliferation, and osteodifferentiation by mimicking the extracellular matrix (ECM) of natural bone tissue [13–15]. Scaffolds with pores diameter of 100–300 µm enable successful diffusion of essential nutrients and oxygen for cell survival, and efficiently regulate the differentiation of stem cells [16–19]. Mesenchymal stem cells (MSCs) can differentiate into numerous categories of cells, which include adipocytes, osteocytes, fibroblasts, and chondrocytes. MSCs have been widely studied compared to other stem cell types for the development of engineered tissue/cell-based therapies [20, 21]. The adipose tissue is the richest source of MSCs in adults, easily accessible [22]. Adipose tissue-derived MSCs lack of phenotypic characterization but they are marked by a low immunogenicity [23]. These cells are designed at the molecular level with immunophenotype properties [17]. Whereas the choice of a proper biomaterial for a three-dimensional scaffold fabrication is crucial in stimulating bone regeneration [24, 25]. Scaffolds are designed to avoid immunological rejection and make them biocompatible, biodegradable, and regulate cell proliferation and differentiation, by controlling their physico-chemical properties [26, 27]. Natural and synthetic polymers have been widely used as biomaterials due to their unique properties such as porosity, pore size, biokinetics, physicochemical, and mechanical properties. HA is widely used as promising osteogenic biomaterial, due to its chemical and structural similarity with mineral phase of bone ECM, along with slow biosorption [28]. Among synthetic polymers, poly-caprolactone (PCL) is a semi-crystalline polyester widely used as biomaterials in medical applications. PCL has a low melting point (55 °C) and suitable properties (porosity, degradation time, and bioreabsorption) for bone tissue regeneration. PCL has a poor wetting surface and establishes weak interactions with biological fluids, preventing cell adhesion and proliferation. For this reason, HA has been incorporated in PCL matrix enhancing mechanical properties and osteogenic features of final PCL/HA scaffolds [29, 30]. This review article aims at providing a brief overview on PCL/HA composite scaffold fabrication techniques such as sol–gel, rapid prototyping, electro-spinning, particulate leaching, thermally induced phase separation (TIPS), and freeze-drying, as suitable approaches for tailoring morphological, mechanical, and biodegradability properties of the scaffolds for bone tissues, but cannot be completely controlled through these methods. Among these methods, the 3D plotting method shows improvements in pore architecture, mechanical properties, biodegradability, and good bioactivity in bone tissue regeneration.

## Methods involved in the fabrication of 3D scaffolds

### Solvent casting and particulate leaching

**Fig. 1:**
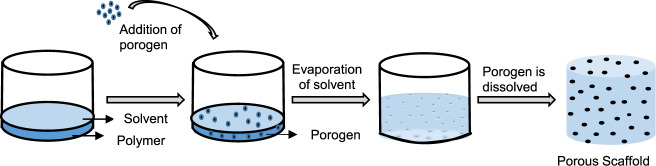
Scheme of composite scaffold by solvent casting and particulate leaching fabrication method

Particulate leaching method, involving a polymer solution incorporated with salt particles of a known diameter, still attracts great interest with the aim of developing optimal porogen with paraffin beads [31], sucrose, and sodium bicarbonate as ingredients [32]. Salt, sugar, glucose, gelatin, and ammonium chloride are used to produce the pores [33–40]. Microporous structure with the pore size of 300–350 μm is formed, after soaking the scaffolds in water, where the porogen (250 μm) dissolves completely leaving behind the empty spots corresponding to pores (Fig. 1) [41]. Fabbri et al., reported a combination of sodium chloride and sodium bicarbonate as porogen, to develop a PCL/HA composite scaffold showing a 70–80% porosity, good interconnectivity, and improved mechanical properties [42]. Zhu et al. prepared PCL/HA scaffolds with more than 93% porosity and 500 μm pore diameters. They were able to control the porosity based on the shape and the amount of the porogens added [43]. With the availability a wide range of porogens, it is possible to generate pore sizes of 50–400 μm with a reasonable degree of control. The scaffolds have interconnected pore structures with a pore size of 600 μm [44]. The pore size of the PCL/HA scaffold was similar to the human trabecular bone (300–1000 μm). Thadavirul et al. reported that PCL/HA composites treated with NaOH, showed a well-defined interconnected pores, increased cell proliferation, high water absorption capacity, high bone matrix deposition, and improved hydrophilicity of the scaffold [45]. A systematic study has been performed, by loading three different HA concentrations (13, 20, and 26 vol %) in PCL-based composites, for improving the mechanical properties [46]. The HA nanoparticles incorporation within PCL composites increases cell differentiation, but it decreases the porosity and cell proliferation in the scaffolds [44]. In 14 days of cell culture, MTT assay, Alkaline phosphatase (ALP) activity for cell differentiation and total protein content were measured [47]. Compared to HA (13, 20, and 26 vol %), PCL/HA (90/10 vol%) showed 85% porosity with the tensile modulus of up to ±28 MPa and, increased in compression modulus and stress upto ±30 and 15.6 MPa, respectively [42]. Guarino et al. described of MSCs seeded onto porous PCL/HA composites scaffolds and cultured in an osteogenic medium for 1–5 weeks. In 3–4 weeks, MSCs were able to adhere and grow on composite substrates, but the small effect of signals on the biological response was evaluated in MSC cell culture. To overcome this problem, a pre-osteoblastic cell line (MC-3T3-E1) has been used, showing a better cell adhesion and enhanced pre-osteoblast response on PCL/HA 3D scaffolds developed by this method. Porous PCL/HA composites are potential biomaterials for bone substitution, which exercise a beneficial influence on structural characteristics [46]. Compared to the other methods, scaffolds with interconnected networks, defined pore size, and increasing porosity can produce a greater degree of control so that the mechanical strength and the biological response can be maximized [48, 49]. Although the leaching method has defined shape, salt particles led to poor interconnection during the scaffold fabrication, and this may not provide optimal scaffold permeability in vitro for cell distribution.

### Sol–gel method

**Fig. 2:**
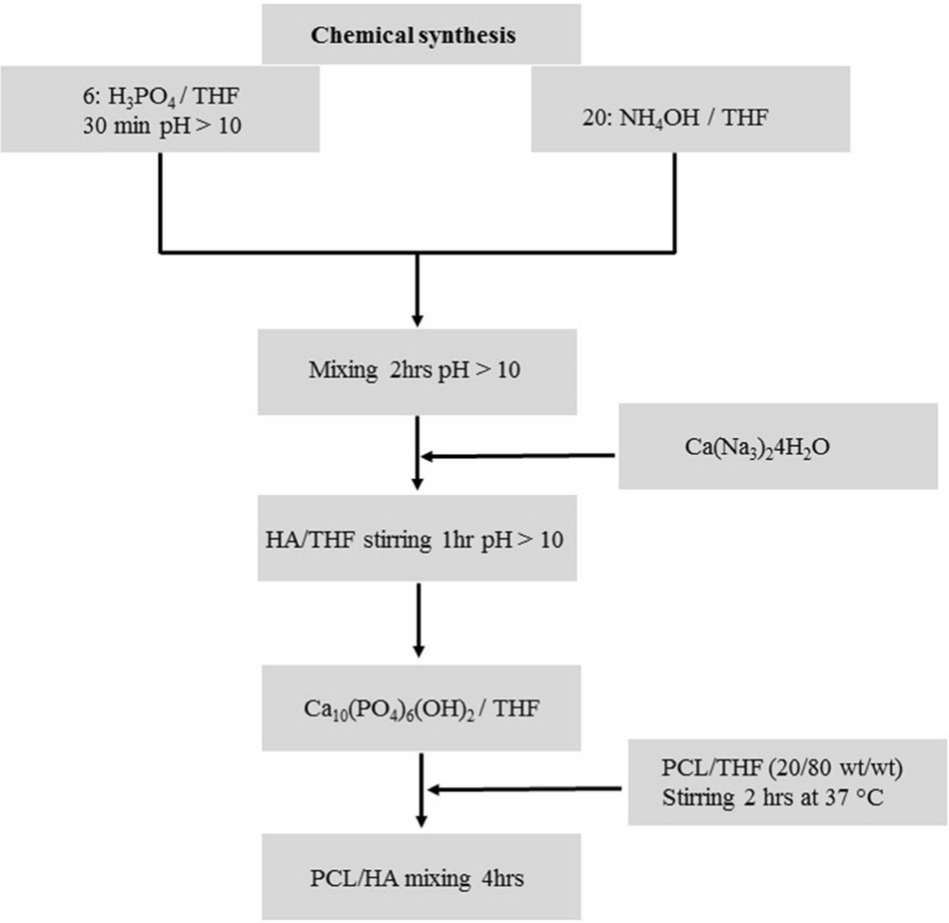
Flow chart of PCL/HA scaffold preparation by chemical synthesis

In the sol–gel process, the particle size is directly controlled by the interaction between calcium and phosphate with non-alkoxide, calcium nitrate tetrahydrate, ammonia, and phosphoric acid as precursor materials under controlled temperature and pH conditions (Fig. 2) [50–52]. Raucci et al. synthesized composite biomaterial made up of HA/PCL (25/75 and 40/60 w/w) by sol–gel method using calcium nitrate tetrahydrate and di-phosphorous pentoxide as precursors, with ethanol and tetrahydrofuran as the solvent at room temperature. Further, the addition of salt as a porogen with a defined size range (210–300 μm) showed a porous structure of 88%. Later, a composite scaffold (PCL/HA) was treated in the simulated body fluid (SBF) in a controlled pH (6.5) environment and observed under SEM [53]. It forms apatite from the distribution of HA particles in the scaffold and the coating presence was stained with 0.5% trypan blue. The composite scaffold improves the hMSC differentiation towards the osteoblast phenotype observed in ALP activity [54]. Costa et al. studied HA nanorods synthesized by a sol–gel-hydrothermal process, which used amorphous calcium phosphate as a precursor material. PCL/HA composites were produced by a mixture of HA powders with PCL resulting in a product of nanorod and nanowires HA/PCL scaffold. The composite scaffold was characterized by SEM, XRD, and FTIR and showed uniform distribution of HA nanorods within the PCL matrix. HA nanorods incorporated within PCL composites significantly increase the mechanical properties of Young’s and compressive modulus from 193 to 665 MPa and 230 to 487 MPa respectively. The HA nanowires with PCL composite can be used for bone tissue engineering [55].

### Freeze drying

**Fig. 3:**
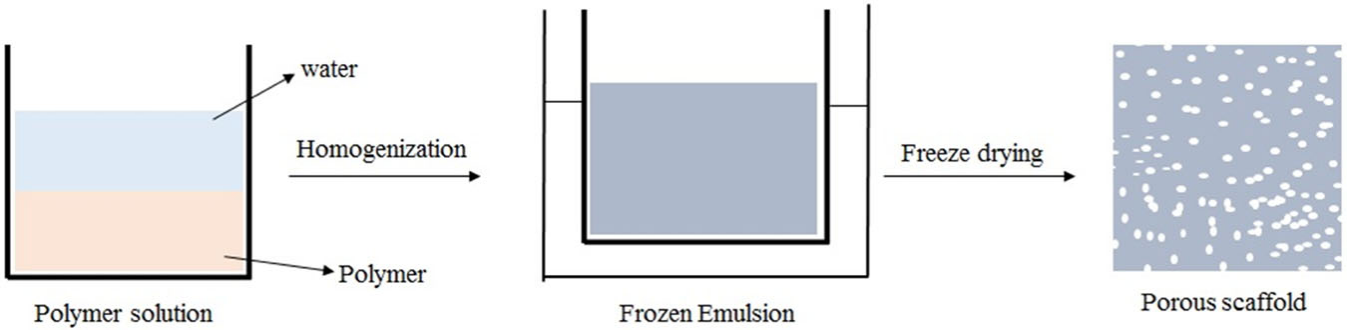
Scheme representation of composite scaffold by freeze-drying method involves the preparation of an emulsion created by homogenization of a mixture of the polymer solution, and a water phase, where the continuous phase has the polymer-rich solvent and the dispersed phase is water, quickly cooling the emulsion to catch in the liquid phase construction, and eliminating the solvent and water by freeze-drying

Freeze-drying is a widely used conventional method for the fabrication of 3D scaffolds, making the solution to freeze at low temperature (−70 to −80 °C), over the primary drying process in which the pressure is lowered through a partial vacuum in the chamber, while water and solvent in the material are removed by sublimation (Fig. 3) [56–59]. Jain et al. used chitosan, PCL, and HA to fabricate porous 3D scaffolds to achieve a pore size ranging between 50 and 200 μm, and 90% of porosity. These composite scaffolds enhanced the mechanical properties, cell proliferation, differentiation, biodegradability, and improved osteoconductivity [60–63]. Choi et al. produced PCL/HA porous scaffolds using freezing drying methods with different HA contents (0, 5, 10, and 20 wt %). The HA particles uniformly distributed in the PCL matrix showed significant improvement in pore size, porosity, and mechanical properties (Table 1). The MC3T3 cells were seeded on the porous scaffold with different HA contents. After day 1, PCL/HA scaffolds showed that the MC3T3 cells attached themselves on the surface and differentiated there. Still, the highest cells proliferation was observed in composite PCL/HA scaffolds with 20 wt% HA [64]. Sharon et al. incorporated the composite PCL/HA scaffold with conductive polymer polypyrrole (PPY) to increase the pore size from 50 to 250 μm and the average pore size was 130.4 μm compared to PCL and PCL/HA scaffolds which had an average pore size 123.7 and 91.6 μm respectively. Also, composite PCL/HA/PPY scaffolds improved mechanical strength, biodegradability, and mediated electrical stimulation to enhance bone regeneration [65, 66]. Although scaffold porosity increases, the mechanical properties decreases. To improve the mechanical strength Hamlekhan et al., studied composite PCL/HA/Gelatin prepared by solvent casting combined with the freeze-drying method, showing that the scaffolds were mechanically effective due to an increase in the stress, stiffness, and elastic modulus (Table 2). SBF and the cytotoxicity studies reported the gradual development of the apatite layer and biocompatibility. These results indicated that the fabricated scaffold possesses the prerequisites for forming/acting as substitute for bone tissue [67].

**Table 1:** Pore size, porosity, and compressive modulus of PCL/HA composite scaffolds produced with various HA contents (0, 5, 10, and 20 wt %) [64]

HA content (wt. %)	0	5	10	20
Pore size (µm)	9.2 ± 0.7	8.5 ± 0.4	6.1 ± 0.7	4.2 ± 0.8
Porosity (vol %)	83.0 ± 0.6	83.9 ± 0.2	84.5 ± 0.2	85.0 ± 0.6
Compressive modulus (MPa)	0.1 ± 0.02	1.2 ± 0.07	2.1 ± 0.06	2.7 ± 0.08

**Table 2:** Mechanical properties of the composite Scaffolds GEL/HA with a different weight percentage of PCL content [67]

PCL content (wt. %)	0	20	30	40	50
Elastic modulus (MPa)	8	16	19	20.5	23.5
Stress (MPa)	1.83	3.13	3.40	3.71	3.73
Stiffness (K/mm)	38	79	93	114	131

### Thermally Induced phase separation (TIPS)

**Fig. 4:**
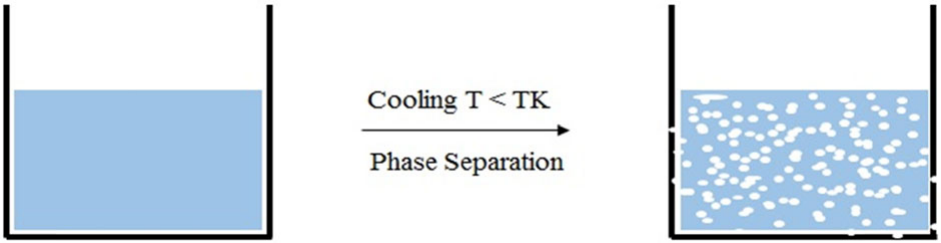
Scheme composite scaffold by thermal induced phase separation method (TIPS) for scaffolds designing consists of quenching the polymer solution under the solvent’s freezing point (Tk) and enforcing liquid–liquid separation to shape two phases: a polymer-rich phase and a polymer-poor phase. The polymer-rich phase solidifies and the polymer poor phase crystallizes. Subsequent removal of crystals by sublimation leaves a porous polymer scaffold

TIPS process consists of quenching the polymer solution under the solvent freezing point and enforcing liquid–liquid separation (Fig. 4) [68, 69]. In this method, HA nanoparticles were incorporated in the PCL polymer solution to make composite scaffolds (PCL/HA). HA nanoparticles were uniformly distributed in the PCL polymer matrix and observed by SEM. The PCL/HA scaffold shows an increase in the compressive modulus of 4.20 MPa and a decreases in the porosity of the structure (70%), after HA nanoparticles incorporation [70]. Guan et al. reported that increasing the concentration of the polymer solution decreases the pore size and porosity of the final 3D composite scaffold [71]. PCL/HA composite scaffold was kept at −18 °C for improving pore size distribution, which can be controlled by solvent phase crystallization. The HA/PCL composite scaffolds showed good osteoconductive property for bone tissue engineering applications [70]. To increase the pore architecture, Salerno et al., dissolved the polymer in an ethyl lactate/water mixture, with salt as porogen, increasing the interconnectivity and achieving a 92% of porosity [72].

### Electro-spinning method

**Fig. 5:**
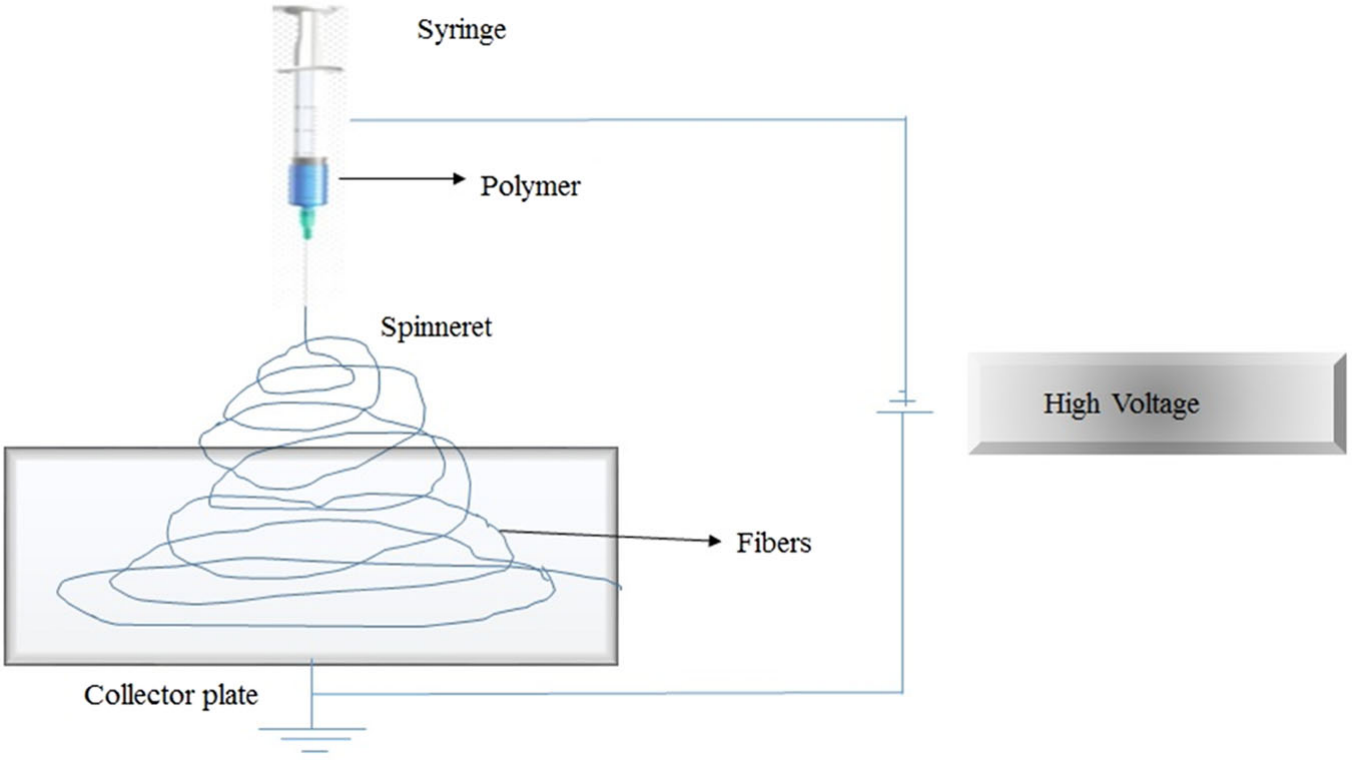
Scheme of electrospining process for composite scaffold fabrication through fiber deposition

The electro-spinning technique is a simple method for 3D scaffold fabrication, based on fibrous structure, able to mimic natural ECM with an interconnected pore structure. The method helps to control mechanical properties and ensures increasing porosity (Fig. 5) [73, 74]. Wutticharoenmongkol et al. fabricated 12% w/v PCL fibrous scaffold with different HA nanoparticles content (0.5 and 1.0%) by electrospinning. The fabricated fibrous scaffold showed an increased porosity of 82 and 90%, while the pore size ranged from 4.3 and 5.6 μm. The tensile strength of the fibrous scaffold increased to 3.6 and 3.8 MPa with respect to HA content (0.5 and 1.0% w/v). Human osteoblasts (SaOS2) cells were cultured on the fibrous scaffold, and the result of cell viability (MTT assay) together with cell differentiation (ALP assay), suggested the biocompatiblity and stimulate cell differentiation [75]. Polini et al., found that the incorporation of either HA or TCP into the PCL nanofibers supports bone mineralization, cell viability, and cell growth. Quantitative analysis of mRNA expression on Runx-2 resulted in a strong stimulation of osteogenic differentiation; and bone sialoprotein was associated with bone mineralized tissue differentiation, in the absence of osteogenic supplements. The nanofibrous structure and the chemical composition of the scaffolds regulate the hMSCs differentiation [76]. In order to increase the mechanical properties of scaffolds, Catledge et al., designed a novel triphasic nanofibrous scaffold from a mixture of PCL/nHA/Collagen ratio of 50*/*30*/*20, respectively. The triphasic scaffold stained with calcein showed uniform distribution of bone-like apatite particles on the polymer matrix with agglomeration. The triphasic scaffold improves the mechanical properties of Young’s modulus by 2.7 GPa [77]. Silicate-containing hydroxyapatite (SiHA) is widely studied for the fabrication of scaffolds, which may improve the bioactivity and osteogenic potential [78, 79]. Shkarina et al., designed and fabricated 3D composite scaffolds (SiHA/PCL) with a predetermined fibers orientation, randomly oriented (rPCL-SiHA) and well-aligned (wPCL-SiHA), to mimic ECM and characterized by synchrotron μCT. A significant increase in the MSCs proliferation and differentiation on wPCL-SiHA, rather than rPCL-SiHA, was observed after 10 days of cell culture [80].

### Rapid prototyping

Rapid prototyping technique (RP) is an advanced technique for the fabrication of well-designed 3D scaffolds with the interconnected porous structure associated with biomolecules and cells [81, 82]. This techniques can fabricate composite PCL/HA scaffolds for repairing damaged bone, specifically, in the analysis of the mechanical properties [83]. Powder-based 3D printing (3DP) is an alternative method to fabricate scaffolds, but the poor mechanical properties restrict their application in bone tissue engineering. Kim et al., prepared calcium sulfate hemihydrate scaffold printed using a 3DP. The fabricated 3DP calcium sulfate hemihydrate powder was transformed into HA upon being treated with ammonium phosphate solution. The scaffolds were coated with PCL polymer solution (5 and 10% w/v) to significantly increase the mechanical strength to 46.86 ± 1.19 and 87.96 ± 6.05 MPa respectively and decrease the porosity of material compared with 5% PCL coated scaffolds. MG-63 cells cultured on 10% w/v of PCL coated HA loaded scaffold showed an increase in osteogenic genes expression, namely Col1A1, Runx2, OSX, and OC, as revealed by semi-quantitative RT-PCR analysis. MTT and ALP studies reported improved the cell attachment and proliferation on 10% w/v PCL coated scaffold [84]. To increase the porosity of the scaffolds. Park et al., designed and fabricated composite scaffolds with a shifted pattern structure (PCL/HA/SP) by 3D plotting system to improve cell adhesion. The PCL/ HA/SP scaffold shows a good interconnected network, highly regular pore size higher than 600 μm, and porosity of 92% with well-defined geometry. The scaffold with shifted pattern had denser structure than PCL/HA and PCL. MTT assay and ALP activity resulted in an increase of cell proliferation and differentiation in PCL/HA/SP compared to the PCL and PCL/HA scaffolds. The mechanical modulus of PCL/HA/SP is not significantly higher than the PCL and PCL/HA scaffolds [83]. Shigang Wang et al., fabricated PCL/HA composite scaffold with a porous circular structure by using 3D printing technology [85]. To further improve the mechanical properties, Liao et al., fabricated the triblock polymer mPEG-PCL-mPEG (PCL) scaffold and mPEG-PCL-mPEG/HA (PCL/HA) by solid free fabrication method [86]. The HA powder with a size of 100 μm was incorporated with mPEG-PCL-mPEG (PCL) by 0.5 weight ratio. PCL/HA biocomposite scaffold showed an increase in pore size, porosity, and mechanical properties of 374.32 ± 11.25 μm, 80%, and 18.38 MPa, respectively [87]. In a similar study by Kim et al., HA/PCL produced composite scaffold by NIPS method, with HA particles dispersing uniformly in a PCL solution induced by the exchange of the tetrahydrofuran and ethanol [88–93]. The process solidifies PCL/HA filaments to create the porous PCL/HA composite scaffold [94]. These PCL/HA filaments were constructed by 3D design in a layer-by-layer separated by highly porous material. The addition of HA particles to the PCL polymer has shown significant increase in mechanical properties, and the ability to form apatite. The MTT and ALP studies have shown an increase in cell proliferation and differentiation of PCL/HA scaffolds [95]. Table 3 shows the morphological, mechanical, and compositional features of PCL/HA-reinforced scaffolds fabricated by employing different additive manufacturing techniques: FDM, DIW, SLS, and 3D printing [96]. Advantages and disadvantages of fabrication methods applied for generation of porous composite scaffolds PCL/HA are shown in Table 4.

**Table 3:** HA constructs processed by additive manufacturing techniques

AM techniques	Porosity (%)	Compressive strength (MPa)	Yield strength (MPa)	Wt.% of HA	Ref.
FDM	26	15	80	30	[97]
DIW	–	24 ± 5	110 ± 20	70	[98]
SLS	37	3.2	67	30	[99]
3D printing	–	7	40	(0:100–50:50)	[100]

**Table 4:** Advantages and disadvantages of the different fabrication methods for composite (PCL/HA) scaffolds fabrication, intended for bone tissue engineering, reported in this review

	Scaffold name	Porosity and size	Advantages and disadvantages	Ref.
Conventional methods	Salt and particulate leaching	>90%, >500 μm	+; Porosity and pore size may be controlled by the size of porogens, Easy to process, no heat. −; Interconnectivity network may not be controlled, longer period, possible toxic solvent residues	[101–103]
	TIPS	>90%, 200 μm	+; High porosity, pores may be controlled. −; Create small pore size, porous structure of scaffold cannot be Controlled, toxic solvent be used.	[68, 69]
	Sol–gel	88%, 300 μm	+; Porosity, pore size can be controlled. −; Longer time to precipitate/gel, toxic solvent be used.	[50, 51]
	Freeze drying	85%, 250 μm	+; High porosity, pore architecture of the scaffold may be controlled by phase separation conditions. −; Utilizes high energy, toxic solvent be used.	[56, 104]
Nanoscale method	Electrospining	>82%, 4.3–5.6 μm	+; Micro or nanoscale fibers with wide range of pore size distribution, high surface area and high porosity. −; Low mechanical strength because of size of fibers.	[105]
Solid freeform fabrication method (SFF)	3D printing	92%, 600 μm	+; Pore size, porosity and interconnectivity can be precisely controlled.Porosity and pore size −; Specific material properties needed.	[106]

+: advantages, −: disadvantages

## Conclusions

Tissue engineering emerges as a promising strategy to overcome the challenges of conventional bone graft treatments. PCL-based composite will be a promising biomaterial for bone tissue engineering. The combination of PCL with HA nanoparticles can result in 3D scaffold as suitable bone graft substitutes, owing to their properties such as mechanical strength, pore architecture, osteoconductivity, and osteoinductivity. PCL/HA composite scaffolds are non-toxic for cells and enhance the slow degradation, which may be suitable for bone tissue engineering. The conventional fabrication methods such as sol–gel, solvent casting, and particulate leaching, electro-spinning, freeze-drying, and TIPS cannot control the pore architecture, geometry, or pore distributions of the scaffolds. On the contrary, rapid prototyping methods have been introduced to overcome the problem of conventional methods, developing customized scaffolds with high porosity, pore architecture control, ideal geometrical shape, mechanical properties, internal morphology, and mass transport properties. Specifically, the rapid prototype methods are able to fabricate the scaffolds in a layer by layer, starting from a 3D computer model of the scaffold designed with specific characteristics. The 3D plotting method is useful in the fabrication of porous PCL/HA composite scaffolds with controlled micro/macro-porous structure, mechanical properties, bioresorption along with inherent osteogenic features, which are crucial for bone tissue regeneration.
